# Single-Cell Immune Atlases to Map Small Extracellular Vesicle Cargo in Tuberculosis–Diabetes Comorbidity: A Narrative Review and Conceptual Roadmap

**DOI:** 10.3390/ijms27083437

**Published:** 2026-04-11

**Authors:** Ramona Cioboata, Silviu Gabriel Vlasceanu, Denisa Maria Mitroi, Anca Lelia Riza, Mara Amalia Balteanu, Oana Maria Catana, Mihai Olteanu

**Affiliations:** 1 Department of Pneumology, University of Medicine and Pharmacy, 200349 Craiova, Romania; ramona_cioboata@yahoo.com (R.C.); mihai.olteanu@umfcv.ro (M.O.); 2 Department of Pneumology, Victor Babes University Hospital, 200349 Craiova, Romania; 3 Department of Physiology, Carol Davila University of Medicine and Pharmacy, 050474 Bucharest, Romania; silviu.vlasceanu@drd.umfcd.ro; 4 Department of Thoracic Surgery, Marius Nasta Pneumology Institute, 050159 Bucharest, Romania; 5 Doctoral School, University of Medicine and Pharmacy, 200349 Craiova, Romania; denisa_maria2@yahoo.com (D.M.M.); oana_cattana@yahoo.com (O.M.C.); 6 Laboratory of Human Genomics, University of Medicine and Pharmacy of Craiova, 200638 Craiova, Romania; 7 Department of Pulmonology, Faculty of Medicine, Titu Maiorescu University, 031593 Bucharest, Romania

**Keywords:** tuberculosis–diabetes comorbidity, TB-DM, extracellular vesicles, EV, single-cell RNA, sequencing, immunometabolism, biomarkers

## Abstract

Tuberculosis–diabetes mellitus (TB-DM) is increasingly recognized as a syndemic in which chronic metabolic dysregulation amplifies tuberculosis severity, delays treatment response, and increases relapse and mortality. However, conventional systemic correlates soluble cytokines and bulk whole-blood transcriptomic signatures often appear broadly similar between TB and TB-DM. This highlights a key gap: clinically meaningful immune dysfunction in TB-DM likely resides in specific lung and blood cell states that are poorly resolved by bulk assays. Small extracellular vesicles (EVs) in plasma and bronchoalveolar lavage (BAL) provide a tractable “liquid biopsy” layer because their RNA and protein cargo can integrate information from infected macrophages, neutrophils, and epithelial/endothelial compartments, and may also include pathogen-derived components. Yet most EV studies remain bulk and cell-agnostic, and interpretation is constrained by heterogeneous vesicle mixtures, selective cargo packaging, and co-isolated non-vesicular contaminants, issues that are especially problematic for nucleic-acid claims without rigorous controls. In this targeted narrative review (2010–2026), we argue that single-cell and multimodal immune reference atlases, including scRNA-seq/CITE-seq, provide a needed scaffold to link EV cargo patterns to specific immune cell states, pathways, and anatomic compartments in TB-DM, enabling prioritized candidates and testable hypotheses. We outline three complementary frameworks: reference-atlas anchoring to project EV cargo modules onto atlas-defined immune states; orthogonal triangulation combining computational inference with immunoaffinity enrichment, targeted validation, and functional assays; and cautious use of “droplet-era” extracellular signals as hypothesis-generating priors for EV-producing states. Implemented in longitudinal, clinically annotated cohorts with standardized EV workflows, atlas-guided EV profiling could yield cell-of-origin–resolved biomarkers of TB-DM immunopathology and treatment response, while prioritizing mechanistically plausible targets for host-directed intervention.

## 1. Introduction

### Clinical Problem and Rationale for Extracellular Vesicle Mapping as the Missing Link

TB–diabetes mellitus (TB-DM) is increasingly recognized as a syndemic immunometabolic comorbidity occurring at the population scale. The WHO Global Tuberculosis Report 2025 estimates 10.7 million incident TB cases in 2024 and 1.23 million deaths (including 150,000 among people living with HIV) [1,2]. Diabetes is also a major driver of TB: ~0.93 million incident TB cases in 2024 (~8.7% of the global burden) are attributed to diabetes, placing dysglycemia alongside undernutrition among leading population-level contributors to TB disease [3,4]. Clinically, TB-DM is not simply additive: chronic hyperglycemia and dyslipidemia reprogram host metabolism and anti-mycobacterial immunity, contributing to higher bacillary burden, cavitary disease, delayed sputum conversion, relapse, and increased mortality compared with TB alone [2,5]. Mechanistically, diabetes impairs macrophage phagocytosis and antigen presentation [6,7], blunts Th1 immunity, and alters lymphocyte numbers, while sustaining low-grade inflammation and lipid dysregulation that intersect with TB pathology and diabetic complication pathways [8].

Yet conventional systemic correlates only partly explain this excess risk. Cross-sectional cytokine studies report heterogeneous, sometimes elevated and sometimes blunted Th1/Th2/Th17 patterns in TB-DM and pre-DM, without a reproducible profile that tracks cleanly with severity [9,10,11,12]. Likewise, whole-blood/PBMC transcriptomics show that the canonical TB blood signature (IFN- and neutrophil-driven) is largely shared between TB and TB-DM, and robust TB-DM specific classifiers have not generalized across settings, despite evidence that diabetes amplifies inflammatory modules and engages metabolic/vascular programs beyond diabetes alone [13,14]. This gap between similar bulk blood signatures and worse outcomes suggests that tissue and cell-state-specific immune defects, macrophage dysfunction, and deeper immunometabolic rewiring are central drivers of TB-DM pathology [15,16,17].

**Figure 1:**
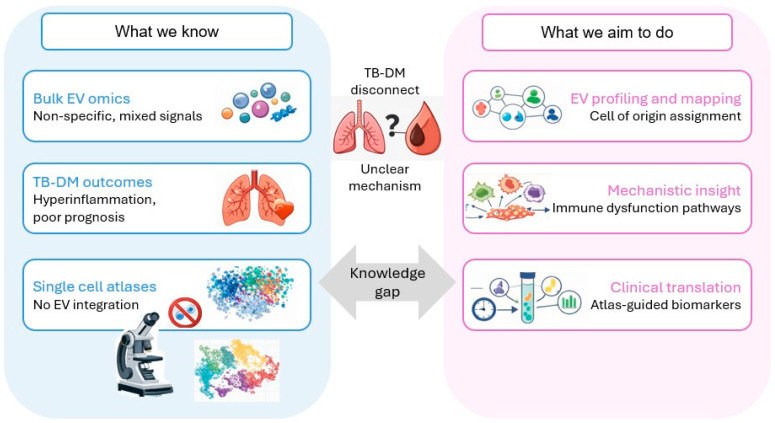
Current gaps and proposed atlas-anchored strategy. TB: tuberculosis, DM: diabetes mellitus, EVs: extracellular vesicles.

A key conceptual and technical limitation is that extracellular vesicles (EVs) and immune cell states are usually profiled in parallel but rarely integrated in a way that links EV cargo to defined single-cell sources and recipient states. Plasma and bronchoalveolar lavage (BAL) EV cargo can now be profiled at depth and used to infer tissue or cellular origin in bulk or probabilistic ways [18,19,20], while scRNA-seq/CITE-seq provides high-resolution maps of immune states and interaction networks [21]. However, EV studies [22,23] often remain “cargo-first” (biomarker or bulk effector framing) without assigning specific EV populations to well-defined immune subsets and programs in the same biological system [24], and single-cell atlases rarely include matched EV measurements to close the loop from cell-of-origin to EV cargo to downstream immune-state effects in vivo [25] (Figure 1).

We propose that single-cell immune reference atlases provide the critical scaffold needed to link EV cargo to defined lung and blood immune cell states in TB-DM. In doing so, bulk plasma and BAL EV profiles can be reframed from non-specific biomarker signals into a cell-of-origin resolved, mechanistically interpretable liquid-biopsy readout of lung immune pathology.

High-quality immune reference atlases demonstrate that curated, transcriptome-defined immune subsets and maturation programs can be used as a template to classify new single-cell data and infer lineage and function, even across tissues and species [26]. Projection and label-transfer algorithms already leverage such annotated atlases to assign cell identities or functional programs to incoming datasets, provided the reference is biologically appropriate and well curated [26]. In parallel, TB-focused studies show that EV-related gene signatures and EV-defined molecular subgroups correlate with distinct immune cell infiltration patterns and immune pathway activation, and can be anchored to cell types using scRNA-seq [27]. More broadly, EV cargo analysis has matured into a scalable, multi-omic technology for disease diagnostics, but remains largely bulk and cell-agnostic, emphasizing biomarker discovery over mechanistic, cell-resolved interpretation [28,29,30].

Bringing these strands together, a TB–DM single-cell immune atlas could serve as a reference space onto which EV transcript/protein cargo from plasma and BAL is computationally projected, allowing probabilistic assignment of vesicles to macrophage, epithelial, endothelial, or specific lymphocyte states and maturation programs [26]. This would close the current gap between EV profiling and immune-state mapping, enabling an atlas-guided “next-generation liquid biopsy” that reports not only that EVs are altered in TB–DM, but which diseased cell states are speaking, what they are saying, and how this communication encodes lung immune pathology [27].

Single-cell atlases evolve from static maps into interpretive engines for EV cargo, turning diffuse plasma/BAL vesicle signatures into a mechanistic, cell-resolved window on TB–DM immunopathology and therapeutic response.

## 2. Search Strategy

**Figure 2:**
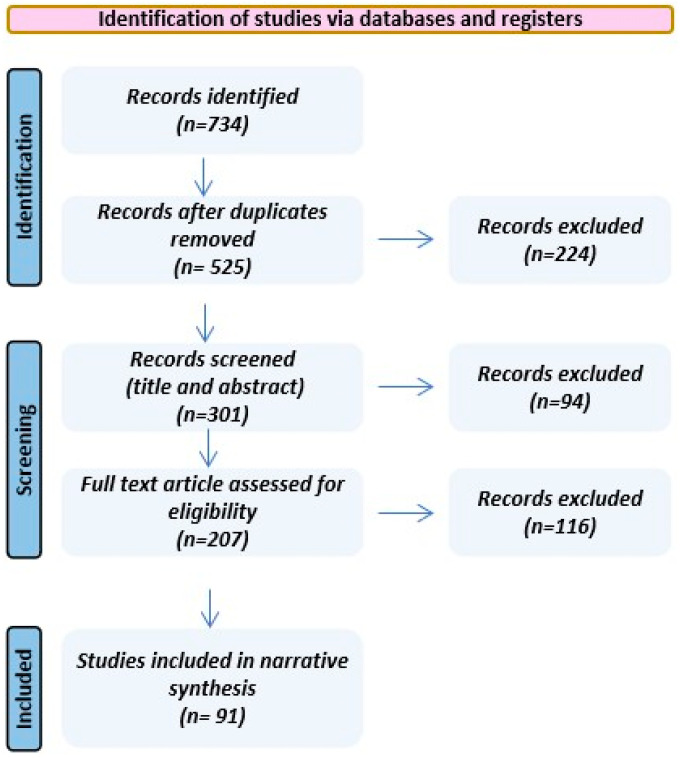
Descriptive flow diagram for study selection.

To support a targeted narrative review, we searched PubMed/MEDLINE, Embase, Scopus, and Web of Science for the English-language literature published between 2010 and 2026. Search terms covered tuberculosis/TB, diabetes mellitus/dysglycemia, extracellular vesicles/small EVs, bronchoalveolar lavage, immunometabolism, biomarkers, and single-cell/multimodal immune atlases (including scRNA-seq and CITE-seq). Titles/abstracts were screened for relevance to TB-DM immunopathology and EV biology, followed by full-text review of prioritized papers. Evidence was synthesized qualitatively based on conceptual and translational relevance rather than formal data extraction; therefore, no risk-of-bias appraisal or meta-analysis was performed. A descriptive flow diagram summarizing the selection process is provided (Figure 2).

## 3. Core Biology Primer: EVs as Immune Communication Units

### 3.1. Extracellular Vesicle Subclasses

Clinical EV samples from blood, cerebrospinal fluid (CSF), urine or tissue are typically heterogeneous mixtures of vesicles. Because current methods cannot cleanly separate exosomes from similarly sized microvesicles, expert guidelines recommend the umbrella term small EV (<~200 nm) in most clinical contexts. We use the operational term ‘small EV’ for <~200 nm preparations and report them by measurable attributes (isolation method, size range, and marker characterization) rather than asserting biogenesis (exosome vs. microvesicle) when purity cannot be demonstrated [31].

EVs are traditionally categorized by biogenesis into exosomes (≈30–150 nm), which arise as intraluminal vesicles within multivesicular bodies and are released upon exocytosis; microvesicles/ectosomes (≈100–1000 nm), which bud directly from the plasma membrane; and apoptotic bodies (ApoEV; ≈50 nm to >5 μm), which are shed during programmed cell death and may encapsulate organelles and nuclear material [32,33]. In clinical biospecimens, however, substantial overlap in size distributions and molecular features means that EV isolates are commonly heterogeneous mixtures rather than discrete, biogenesis-pure classes, an issue amplified in complex biofluids such as plasma or bronchoalveolar lavage [34]. Methodologically, widely used centrifugation and filtration workflows that enrich vesicles below ~200 nm (e.g., the 100,000× *g* pellet or material passing a 0.22 μm filter) cannot reliably discriminate bona fide exosomes from small microvesicles or small apoptotic extracellular vesicles (ApoEVs), prompting major guidelines to recommend operational classification based on measurable properties size range, density, and isolation method rather than inferred origin when purity cannot be demonstrated [35]. This caution is reinforced by biological ambiguity: canonical “exosome markers” including CD9, CD63, CD81, and TSG101 are not uniquely exosomal and can be detected across EV subclasses, making definitive claims of exosome identity difficult to substantiate in patient-derived preparations [36]. From a translational and regulatory perspective, emphasis similarly shifts from strict biogenesis assignment to the generation of a reproducibly isolated, well-characterized, size-defined EV population with documented quality attributes, potency, and safety. Collectively, these technical, biological, and translational considerations support the use of the origin-agnostic term “small EV” for <~200 nm preparations, accompanied by transparent reporting of isolation procedures, size distribution, and marker characterization, thereby minimizing overinterpretation while improving comparability across studies [37,38].

### 3.2. What EV Cargo Types Can and Cannot Reveal

Across major cargo classes, EV profiling can serve as a sensitive readout of cellular state while imposing important limits on what can be inferred mechanistically. EV proteins often reflect the cell type of origin, activation state, and aspects of vesicle biogenesis; for example, small-EV-enriched preparations are frequently enriched in tetraspanins and endosomal sorting complex required for transport (ESCRT) associated components, whereas larger vesicles more commonly contain organelle-associated proteins [39,40]. Accordingly, unbiased proteomics coupled to pathway enrichment can identify dominant functional programs such as immune modulation, adhesion, extracellular matrix remodeling and yield reproducible “fingerprints” that support biomarker development and immunocapture strategies [41]. However, bulk proteomic signatures rarely permit confident assignment of biogenesis class, such as exosome versus microvesicle, given imperfect marker specificity, and protein detection alone does not establish stoichiometry, delivery efficiency, or single-molecule activity at the level of individual vesicles. Small RNAs, particularly miRNAs, but also tRNA fragments, snoRNAs and other species are often selectively enriched relative to producer cells, consistent with regulated sorting, and their stability and low-abundance detectability make them attractive liquid-biopsy candidates; in selected experimental systems, EV-associated miRNAs can modulate gene expression in recipient cells [42]. Yet typical stoichiometric estimates on the order of ~1 miRNA per 1–100 EVs, and ~1 full-length long RNA per ~1000 EVs imply that many vesicles may not carry a given RNA species, while isolation and library-preparation choices can substantially reshape observed extracellular RNA profiles and many detected reads correspond to fragments of uncertain biological relevance; consequently, presence in bulk EV-RNA does not prove effective in vivo delivery or functional impact [43,44]. Long RNAs can mirror and in some cases appear selectively filtered from the source transcriptome and show promise as disease and tissue-associated biomarkers, but short-read sequencing frequently captures fragmented molecules, leaving the prevalence of intact, translation-competent mRNA or functional long non-coding RNAs uncertain, and bulk assays cannot, without stringent controls, distinguish luminal EV cargo from nucleic acids associated with co-isolated particles or ribonucleoprotein complexes [44,45]. Lipid cargo can report on membrane composition and uptake biology such as enrichment in cholesterol, sphingomyelin, glycosphingolipids, phosphatidylserine and, in some contexts, reveal bioactive lipid mediators and metabolic states relevant to inflammation; however, EV lipidomics remains less standardized, is particularly vulnerable to confounding by co-isolated lipoproteins in biofluids, and functional attribution of specific lipid species to recipient-cell phenotypes is often incomplete [46,47]. Taken together, EV cargo profiles are best interpreted as integrated reporters of cellular and disease states and, in select settings, mechanistic effectors while recognizing that analytical methods strongly influence detected cargo, stoichiometry per vesicle is often low, and molecular presence is not equivalent to functional delivery; rigorous contaminant controls, where possible single-vesicle or digital assays, and direct functional tests are therefore essential for causal claims, particularly in heterogeneous clinical samples where low stoichiometry and co-isolated particles can otherwise produce misleading signals.

EV preparations from complex biofluids almost always co-isolate non-vesicular extracellular material, and this is particularly consequential for nucleic-acid claims. Differential ultracentrifugation and many kit-based workflows can co-enrich soluble proteins, lipoproteins, DNA-protein complexes, and other nanoparticles (including exomeres/supermeres) alongside EVs [48,49]. Benchmarking studies that include vesicle-depleted plasma controls have shown that polymer precipitation can yield particle counts that remain high even when vesicles are depleted, consistent with extensive non-vesicular co-isolation under those conditions and underscoring the need for orthogonal EV validation and contaminant reporting when using precipitation-enriched fractions [50,51]. In transfection-derived EV experiments, DNA-transfection reagent complexes can also co-pellet with EV-enriched fractions and may account for apparent “EV-mediated” genetic effects unless density gradients or other orthogonal purification steps are applied.

Single-vesicle and high-resolution analyses further indicate that DNA detected in “EV” pellets can be largely non-vesicular, such as naked DNA or DNA associated with non-EV particles, and that size-exclusion chromatography may incompletely separate cell-free DNA from EV-containing fractions, whereas density-gradient separation improves discrimination between vesicular and non-vesicular components [52]. Together with broader reassessments of extracellular nanoparticle composition and separation limits [53], these findings argue that claims of EV-associated DNA should explicitly demonstrate vesicle association, such as nuclease protection ± detergent, orthogonal fractionation, or immunocapture, and should avoid inferring active DNA export via small EVs when vesicle association has not been shown [54].

## 4. Host- Versus Pathogen-Derived Extracellular Vesicles in Tuberculosis: Mechanistic Pathways and Biomarker Signals

In tuberculosis, EV biology operates along two parallel but interacting “lanes”: EVs released by infected host cells and mycobacterial extracellular vesicles released by M. tuberculosis itself. Host-derived EVs originate from infected macrophages, neutrophils, and other leukocytes and package a mixture of host cargo together with bacterial antigens and nucleic acids, thereby extending immune signaling beyond the initially infected niche [54,55]. Functionally, these vesicles can promote macrophage antimicrobial programs, including autophagy-linked pathways, enhance antigen transfer to dendritic cells, and support Th1 polarization and interferon (IFN-γ)-producing T-cell responses; yet the same communication channel can be context-dependent and “double-edged,” with certain EV cargos dampening IFN-γ-inducible activation pathways and constraining antigen presentation in bystander phagocytes [56,57,58]. Consistent with this immunological relevance, serum EVs from TB patients can induce immune responses that enhance early defense against BCG in vivo, supporting a functional role beyond biomarker association [59]. These host EV footprints are also measurable in biofluids, where proteomic and transcriptomic signatures, including host inflammatory and metabolic pathways, as well as detectable Mycobacterium tuberculosis (Mtb) peptides in circulating EV fractions, support their development as non-sputum biomarkers for disease state and treatment response [60]. In parallel, Mtb produces its own EVs, which are biogenetically distinct from host EVs and are enriched for cell-envelope lipoglycans, lipoproteins, glycolipids, and secreted proteins that function as virulence-associated immunomodulators. EVs potently engage innate sensing often via TLR2-dependent pathways, shape granulomatous inflammation and tissue remodeling, and deliver antigens to antigen-presenting cells; however, they may also transiently suppress T-cell proliferation or cytokine production under high-exposure conditions, consistent with localized immune modulation that could favor persistence [61]. Importantly, careful fractionation studies indicate that host and bacterial vesicles can be separated as largely non-overlapping populations, which is conceptually essential to avoid conflating “host exosomes carrying bacterial cargo” with bona fide bacterial membrane vesicles. Across both lanes, EVs provide a unifying framework in which vesicle-mediated trafficking of antigens, lipids, and nucleic acids contributes to TB immunopathogenesis while leaving quantifiable signatures in accessible specimens supporting biomarker discovery, mechanistic interrogation, and the rational design of EV-informed vaccines and host-directed therapeutic strategies [62].

## 5. Extracellular Vesicles in Diabetes: Altered Abundance and Cargo Shaping Immune, Vascular, and β-Cell Crosstalk

Across type 1, type 2 and gestational diabetes, EV number and cargo are consistently altered, especially miRNAs and inflammatory proteins. Circulating EVs are increased in type 2 DM, driven by insulin resistance, with shifts in insulin-signaling proteins and cytokines. EVs from obese/diabetic models can transfer a “diabetic phenotype”, insulin resistance, and inflammation to healthy cells, while lean EVs can partially reverse it [63,64,65].

DM reshapes both the abundance and the biological content of EVs, creating an immunometabolic signaling network that links chronic low-grade inflammation to functional impairment of innate immunity, endothelial injury, and progressive β-cell stress. In type 2 diabetes, circulating EVs are often increased and show enhanced uptake by leukocytes, where they can reprogram monocyte transcriptional programs toward pro-inflammatory states while concurrently attenuating oxidative-stress pathways, a combination that may compromise microbial killing and phagocytic competence. Hyperglycemia can further imprint EV-mediated crosstalk across compartments: for example, high-glucose–conditioned monocyte EVs can transfer specific miRNAs to endothelial cells, suppressing cytoprotective stress-response pathways and engaging inflammasome-associated signaling, thereby amplifying reactive oxygen species and IL-1β-linked vascular inflammation. In parallel, diabetes is characterized by elevated endothelial-, platelet-, and monocyte-derived EVs and microparticles carrying inflammatory and angiogenic mediators that correlate with insulin resistance and β-cell dysfunction, consistent with EVs as both reporters and propagators of cardiometabolic risk [66]. Functionally, diabetic EVs can directly perturb endothelial integrity, promoting cytoskeletal remodeling and migration, and critically inducing barrier disruption and hyperpermeability through signaling pathways that converge on junctional instability; these effects highlight EV protein cargo as a plausible driver of microvascular complications [67]. At the level of the islet, β-cells respond to ER stress, cytokine exposure, or glucolipotoxic conditions by increasing EV release and remodeling EV cargo, with stressed β-cell EV capable of activating human monocytes (upregulating antigen-presentation and co-stimulatory programs) and inducing pro-inflammatory cytokines that reinforce islet inflammation. In type 1 diabetes, β-cell-derived EVs enriched in autoantigens and immunoregulatory miRNAs provide an additional mechanistic bridge between β-cell stress and autoimmunity by enhancing antigen presentation, promoting T-cell activation, and contributing to β-cell apoptosis [68]. These same processes create biomarker opportunities: EV-enriched miRNA signatures and autoantigen-bearing vesicles have shown promise as early indicators of β-cell stress and incipient disease, in some settings preceding overt hyperglycemia and outperforming total circulating RNA measurements by capturing vesicle-fraction-specific signals. Collectively, diabetes-driven changes in EV biogenesis and cargo across immune, vascular, and β-cell compartments establish a feed-forward loop in which vesicle-mediated communication sustains systemic inflammation and tissue injury while simultaneously offering tractable, circulating readouts for risk stratification, mechanistic phenotyping, and therapeutic targeting [69].

## 6. The TB–DM Immune Landscape: What Single-Cell Studies Tell Us So Far

Single-cell profiling provides the most direct way to test how diabetes reshapes anti-mycobacterial immunity at the level of specific pulmonary and blood cell states, and it also provides the most defensible rationale for using single-cell immune “maps” as a scaffold to interpret extracellular vesicle signals in TB-DM. Direct single-cell evidence in bona fide TB-DM remains limited, but a recent murine lung study supports the concept that diabetes can shift key protective programs while amplifying inflammatory axes, changes that are difficult to resolve using bulk blood signatures alone [70].

In addition to compositional shifts, TB-DM lung immunity is reorganized at the level of cytokine and pathway architecture. In the Chaudhary et al. dataset, diabetic TB lungs exhibited attenuated type II IFN-γ and TNF response programs, consistent with impaired classical macrophage activation and compromised bactericidal effector functions [70]. In contrast, IL-16-linked signaling and Th17-associated programs were relatively amplified, suggesting a state of heightened inflammation that is qualitatively misdirected and does not compensate for diminished Th1-driven control. Notably, transcriptional signatures of hyperglycemia and dyslipidaemia were interwoven with inflammatory gene modules, reinforcing the concept that metabolic perturbations are not ancillary but are embedded within the immune wiring that governs TB control. Collectively, these findings support a model in which diabetes reshapes the lung immune atlas toward delayed recruitment, reduced protective Th1/M1 states, and increased non-protective inflammatory programs (including IL-16 and Th17), thereby directly motivating a cell-state centric, single-cell–anchored strategy to map extracellular vesicle cargo to pathogenic immune subsets in TB-DM [70].

## 7. Bulk TB–DM Transcriptomics and Conventional Immunophenotyping: Complementary Context for Single-Cell–Anchored Mapping

Whole-blood RNA-seq from multi-country cohorts found that the canonical TB transcriptional signature is broadly similar in TB with or without DM, but with a trend toward greater neutrophil and innate pathway activation and upregulation of micro/macrovascular-complication pathways with rising HbA1c [71]. This indicates that DM superimposes vascular and innate-bias signals on a largely preserved TB signature consistent with the DM-biased monocyte and granulocyte programs seen in single-cell work.

Flow-based studies in TB–DM also show reduced total T, CD8^+^ T, B, and NK cell counts, and progressive loss of Mtb-specific IFN-γ/TNF/IL-2 multifunctional CD4^+^ and CD8^+^ memory responses with increasing hyperglycemia [5,72]. While not single-cell, these findings align tightly with the exhaustion and lymphopenia states mapped by scRNA-seq in TB and by scRNA-seq in T2DM.

Collectively, emerging single-cell resources provide a strong rationale for a mapping strategy that links EV cargo to defined cellular sources and immune states in TB-DM. First, a TB-DM lung single-cell atlas directly demonstrates core features of comorbid immunopathology, including an altered Th1/M1 versus Th17 balance, aberrant IL-16-associated signaling, and attenuated IFN-γ and TNF response programs in diabetic TB lungs [70,73]. Second, multiple TB single-cell atlases now delineate the reference “TB landscape” across blood and lung, resolving changes in cellular composition, exhaustion and effector states, granuloma-associated niches, and monocyte differentiation trajectories that are not apparent in bulk profiling. Third, independent single-cell studies in diabetes show that hyperglycemia and insulin resistance reprogram epithelial and myeloid compartments in the lung and reshape peripheral immunometabolic circuits, providing mechanistic priors for how diabetic physiology can distort anti-mycobacterial immunity. Together, these datasets support the use of a single-cell derived systems map as a rigorous scaffold for TB-DM: diabetes can be conceptualized as shifting pre-existing, TB-critical cellular programs spanning Th1 and Th17 axes, NK and monocyte subsets, and epithelial antigen-presenting states along gradients of exhaustion, misdirected inflammation, and impaired epithelial–myeloid function, in ways that can be quantitatively captured and compared across compartments and disease states [74,75].

## 8. Pivot to TB–DM: A Limited but Emerging Single-Cell Evidence Base

In contrast to the extensive single-cell work in TB or diabetes alone, reports that profile immune cells in bona fide DM–TB are still limited but growing.

The most compelling “atlas-like” resource to date is a murine lung single-cell study by Chaudhary et al., which profiled immune compartments enriched for CD3^+^ and CD11c^+^ cells in an M. tuberculosis infection model with diabetes comorbidity [70]. In that system, diabetes was associated with a broad restructuring of pulmonary immune responses, including reduced type II interferon response programs and a relative increase in Th17-associated signatures, consistent with inflammatory rewiring alongside attenuation of canonical protective axes. While this resource is species and compartment-limited and does not directly measure EV release or EV cargo, it provides a concrete example of the type of cell-state shifts that motivate an atlas-guided strategy for interpreting plasma/BAL EV modules in TB-DM [70].

Complementary systems-level human datasets, although not single-cell, map the peripheral immune and inflammatory milieu in TB-DM. Whole-blood transcriptomics plus plasma analytes reveal that TB-DM shares the canonical TB signature but with amplified neutrophil-rich inflammation and activation of pathways associated with diabetic complications and epigenetic reprogramming [76,77]. Flow- and cytometry-based studies further document reduced total T cells, CD8^+^ T cells, B cells and NK cells, and hyperglycemia-linked lymphopenia, supporting a systemic immune-state shift in TB-DM [17].

Taken together, these datasets constitute early immune-state atlases for TB-DM: single-cell resolution in the lung compartment in mice, and high-dimensional but bulk/systemic maps in human cohorts [70,78].

Despite this progress, none of the current TB-DM immune atlases integrate extracellular vesicles as a mechanistic layer. The scRNA-seq lung atlas focuses on cellular phenotypes and cytokine pathways but does not profile EV release, cargo, or vesicle-mediated communication in TB-DM lungs [70]. Likewise, systems immunology and biomarker studies in TB-DM emphasize blood transcriptomes, soluble cytokines, metabolic pathways, and cell subset frequencies without characterizing circulating EV populations [78,79]. More broadly, reviews of immune dysregulation in TB-DM highlight macrophage dysfunction, altered cytokine signaling, and chronic inflammation, and explicitly call for systems biology approaches such as single-cell transcriptomics, metabolic profiling, and epigenetic mapping, but do not yet incorporate EV omics into this framework [80].

This gap creates a clear niche for an integrated EV single-cell mapping approach: existing work already defines cellular and transcriptional states in TB-DM, but how these states are encoded in, and propagated by, EV cargo remains uncharted.

## 9. Methods and Conceptual Roadmap for Mapping EV Signals to Immune Cell States in TB–DM

This section outlines a structured approach to link EV cargo to defined immune cell states and tissue compartments in tuberculosis with comorbid diabetes mellitus, tailored to translational expectations in clinical research.

EVs isolated from plasma or BAL originate from a wide variety of tissues and cell types, including lung epithelial cells, endothelial cells, and multiple immune lineages [55]. In TB, both host- and Mtb-derived EVs carry microbial antigens and host immune regulators that shape macrophage polarization, cytokine production, and granuloma biology, while in DM, circulating EVs reflect systemic metabolic stress and vascular inflammation [59]. TB and DM each reshape both the mixture of EV-producing cells and the molecular cargo packaged into EVs, and their combination is likely to accentuate this complexity.

The central challenge is to map composite EV cargo proteins, RNAs, and lipids to defined immune cell states, such as dysfunctional macrophages, Th17-like T cells, and to anatomical origin, lung versus blood. Doing so would move the field beyond descriptive EV signatures toward a mechanistic readout of how TB-DM associated immune dysfunction is encoded within circulating and lung-proximal EV pools [30]. The roadmap below, therefore, integrates biofluid- and BAL-derived EV isolation with cell-type deconvolution, cargo profiling, and functional perturbation assays to assign EV signals to macrophage, T-cell, and epithelial states across lung and blood compartments in TB–DM [55]. Because EV enrichment workflows can substantially influence apparent particle yield, cargo composition, and contaminant carryover, TB-focused EV studies also need explicit, method-aware quality benchmarks. Recent work comparing EV enrichment approaches for Mycobacterium tuberculosis-derived EVs proposed minimum protein standards to support cross-study comparability and reduce method-driven artifacts, emphasizing that ‘Mtb-EV’ claims should be accompanied by defined protein markers, transparent reporting of isolation steps, and orthogonal validation of vesicle association rather than relying on particle counts alone. Incorporating such minimum standards into TB–DM study design strengthens downstream atlas-anchored interpretation by ensuring that mapped cargo modules reflect reproducible vesicle populations and not isolation-specific background [81].

## 10. Conceptual Frameworks

### 10.1. Framework 1: Reference Atlas Anchoring

Advances in single-cell and multimodal profiling make it feasible to build immune reference atlases that can serve as an interpretive scaffold for EV data. In this approach, scRNA-seq and/or CITE-seq atlases are generated for BAL/lung and PBMCs across TB-only, DM-only, TB-DM, and control conditions, using integrative multimodal methods such as weighted nearest neighbor frameworks to define immune states from joint RNA protein information and resolve subtle phenotypes [82]. State-specific signatures are then derived for candidate pathogenic subsets such as inflammatory monocyte/macrophage programs and Th17-like T-cell states, including both transcript modules and discriminating surface marker panels. In parallel, EV protein and RNA cargo are profiled from matched plasma and BAL. EV cargo modules are subsequently treated as “queries” and mapped onto the atlas-defined immune state space using enrichment, correlation, or projection-based methods to infer which immune states most plausibly contribute to the observed EV signal. Finally, comparative alignment to lung BAL versus blood reference signatures provides a principled though necessarily probabilistic basis to infer anatomical compartment of origin, positioning EV profiles as readable outputs of an established, biologically interpretable immune-state landscape [83,84,85]. However, any such mapping must be interpreted as inherently probabilistic, given selective and non-stochastic cargo loading into heterogeneous EV subpopulations, technical variability in EV-RNA/protein profiling, and the current lack of standardized, compartment-resolved EV reference datasets. A central methodological priority for next-generation TB–DM EV studies is to detect and correct for the ‘selectivity gap,’ namely the non-stochastic and nonlinear relationship between producer-cell abundance and EV cargo due to state-dependent EV release and cargo sorting [86]. Experimentally, the most informative design is matched sampling in the same individuals and timepoints, combining scRNA-seq/CITE-seq (blood and lung/BAL where feasible) to quantify cell-state abundance and activation programs, with EV multi-omics (RNA/protein ± lipid) from plasma and BAL to distinguish ‘more cells’ from ‘more cargo per cell’ [87]. Where feasible, atlas-informed immunoaffinity enrichment of EV subpopulations can reduce mixture complexity and test whether predicted state-linked modules are selectively enriched, while perturbation or calibration experiments under TB- and diabetes-relevant stimuli can provide empirical mappings between cell state and EV cargo. Computationally, EV interpretation should be framed as a generative mixture problem rather than assuming proportionality to abundance, with separate latent terms for cell-state abundance, EV production rate, and cargo-loading strength, enabling nonlinear behavior and improving identifiability in longitudinal and multi-compartment datasets. Finally, any inferred selectivity should be stress-tested using orthogonal validation such as enrichment fractions, nuclease protection controls for nucleic acids, and alternative isolation workflows to minimize artifacts from contaminants or method-specific biases [81].

### 10.2. Framework 2: Orthogonal Triangulation

Origin assignment from bulk EV omics is inherently uncertain, so more defensible attribution should rely on orthogonal triangulation that integrates computational inference with experimental enrichment and validation. First, computational deconvolution can leverage immune-state signatures derived from single-cell atlases to decompose EV transcriptomic and proteomic profiles into estimated contributions from defined cell states and, where possible, tissue compartments, conceptually analogous to tissue deconvolution approaches and emerging EV deconvolution efforts in other disease areas [83,88]. These estimates must be interpreted cautiously because EV packaging is selective and does not necessarily scale with cellular abundance [56]. Second, experimental enrichment can test these inferences by immunoaffinity capture of EV subsets using surface epitopes informed by CITE-seq, such as markers enriched on macrophage-, T-cell-, or lung epithelial-derived vesicles, followed by targeted validation, qPCR/Nanostring for RNAs, and targeted mass spectrometry for proteins to confirm that predicted state-specific cargo is enriched in the captured fraction. Where feasible, functional assays such as macrophage co-culture with enriched EV subsets can provide an additional layer of evidence by determining whether the mapped EV modules elicit the expected immunomodulatory phenotypes [30,89]. In this framework, credible claims about EV cellular origin require convergent computational and experimental support, rather than correlation-based interpretation alone.

### 10.3. Framework 3: “Droplet-Era” EV Signals

**Figure 3:**
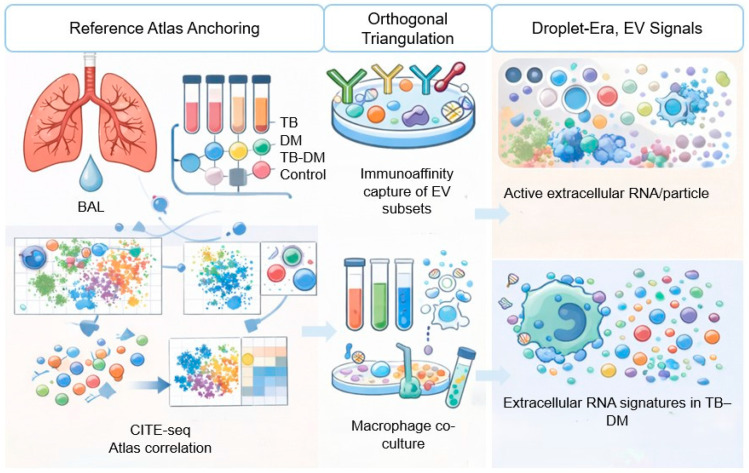
Validation frameworks for linking EV cargo to immune cell states in TB-DM. The figure summarizes three complementary, convergent strategies. Framework 1: Reference-atlas anchoring—matched scRNA-seq/CITE-seq data from lung/BAL and blood define atlas-derived cell states and state-specific signatures, which are used to computationally map bulk EV cargo modules to candidate producing cell types and immune programs across compartments. Framework 2: Orthogonal triangulation—atlas-informed predictions are tested experimentally by enriching candidate EV subpopulations, followed by targeted orthogonal validation of predicted cargo, and, where feasible, functional assays in relevant recipient cells to assess predicted immunomodulatory effects. Framework 3: “Droplet-era” extracellular signals—ambient/extracellular RNA signals observed in droplet-based scRNA-seq are treated as hypothesis-generating priors that can prioritize candidate EV-producing states or niches, but require strict controls and orthogonal validation because they are not EV-specific.

Droplet-based single-cell RNA-seq workflows contain an extracellular component that is often modeled as “ambient RNA” background. While much of this signal reflects technical contamination and RNA released from stressed or lysed cells, the ambient pool may also include transcripts associated with extracellular particles, including RNA carried by extracellular vesicles. Conceptually, this raises a hypothesis-generating possibility: under certain conditions, state-specific patterns in the ambient fraction could report a vesicle-rich microenvironment and thereby provide indirect evidence of active extracellular RNA/particle release in situ. If robust and reproducible, EV-aware modeling of ambient signals could be used to refine priors in atlas-anchored EV mapping by highlighting candidate cell states most strongly associated with extracellular RNA signatures in TB-DM. However, these approaches are technically nascent, are not specific for EV, and require careful controls and orthogonal validation to distinguish vesicle-associated RNA from cell lysis and other sources of extracellular material. As such, “droplet-era” extracellular inference should be positioned as a forward-looking complement to, rather than a substitute for, direct EV profiling and experimental validation [90] (Figure 3).

## 11. Limitations

This targeted narrative review synthesizes a heterogeneous body of evidence and, by design, did not undertake formal risk-of-bias assessment or meta-analysis. Study-level heterogeneity and residual confounding limit causal inference across several domains. Interpretation of plasma and BAL small EV in TB–DM is constrained by several factors. First, EV isolation from clinical biofluids is imperfect: non-vesicular particles and protein/RNA complexes can co-isolate, and pre-analytical variability can shift apparent abundance and cargo, especially for nucleic-acid readouts. Mapping bulk EV cargo to a producing cell type/state is inherently probabilistic. EV packaging is selective and does not scale linearly with producer-cell abundance, so “cell-of-origin” assignments should be treated as hypotheses that require orthogonal validation. Compartment specificity is limited in plasma-first studies because plasma EV integrates signals from many tissues; BAL is more lung-proximal but heterogeneous and sampling-dependent. Finally, the human TB-DM single-cell evidence base is still relatively sparse and heterogeneous across populations, comorbidities, and treatment timepoints, which may limit atlas portability and the generalizability of inferred EV signatures.

## 12. Future Directions

A practical route forward is to treat TB-DM single-cell immune atlases as an interpretive reference space for EV cargo. By generating matched lung/BAL and PBMC single-cell maps across TB-only, DM-only, TB-DM and control states, and projecting plasma/BAL EV multi-omic signatures into this atlas-defined immune-state landscape, bulk EV measurements can be converted from anonymous signatures into probabilistic readouts of specific producing cell states and programs.

Because cell-of-origin inference from EV omics is inherently uncertain and EV packaging is selective, credible attribution will require orthogonal triangulation: computational mapping should be paired with immunoaffinity enrichment of candidate EV subsets, targeted validation assays, and, where feasible, functional perturbation experiments that test whether enriched vesicle fractions reproduce predicted immunomodulatory effects.

Near-term priorities for the field include standardizing EV isolation and reporting with explicit controls for non-vesicular material, building well-powered, clinically annotated TB-DM single-cell reference atlases across compartments and treatment timepoints, and developing mapping models that explicitly account for selective cargo packaging and mixed cellular contributions. Implemented in longitudinal cohorts, an atlas-guided EV “next-generation liquid biopsy” could provide a mechanistically interpretable window into TB-DM immunopathology tracking shifts in macrophage activation programs, Th1/Th17 balance, epithelial–endothelial injury, and treatment response while also identifying EV cargo modules that are plausible mediators of dysregulated immune–metabolic crosstalk and therefore candidates for host-directed intervention

## 13. Conclusions

TB-DM comorbidity presents a population-scale syndemic in which clinical outcomes are consistently worse than TB alone, yet conventional soluble cytokine measurements and bulk blood transcriptional signatures only partially explain the excess risk. Small extracellular vesicles offer an attractive, scalable non-sputum measurement layer because their protein and RNA cargo can integrate information from infected lung immune cells, stromal compartments, and systemic metabolic tissues. However, most EV biomarker studies remain bulk and cell-agnostic, and EV isolates are vulnerable to non-vesicular contaminants that can drive artifactual mechanistic claims if not rigorously controlled.

## Data Availability

The original contributions presented in this study are included in the article. Further inquiries can be directed to the corresponding authors.
